# Continuity of Cancer Care: The Surgical Experience of Two Large Cancer Hubs in London and Milan

**DOI:** 10.3390/cancers13071597

**Published:** 2021-03-30

**Authors:** Maria J. Monroy-Iglesias, Marta Tagliabue, Harvey Dickinson, Graham Roberts, Rita De Berardinis, Beth Russell, Charlotte Moss, Sophie Irwin, Jonathon Olsburgh, Ivana Maria Francesca Cocco, Alexis Schizas, Sarah McCrindle, Rahul Nath, Aina Brunet, Ricard Simo, Chrysostomos Tornari, Parthi Srinivasan, Andreas Prachalias, Andrew Davies, Jenny Geh, Stephanie Fraser, Tom Routledge, RuJun Ma, Ella Doerge, Ben Challacombe, Raj Nair, Marios Hadjipavlou, Rosaria Scarpinata, Paolo Sorelli, Saoirse Dolly, Francesco Alessandro Mistretta, Gennaro Musi, Monica Casiraghi, Alessia Aloisi, Andrea Dell’Acqua, Donatella Scaglione, Stefania Zanoni, Daniele Rampazio Da Silva, Daniela Brambilla, Raffaella Bertolotti, Giulia Peruzzotti, Angelo Maggioni, Ottavio de Cobelli, Lorenzo Spaggiari, Mohssen Ansarin, Fabrizio Mastrilli, Sara Gandini, Urvashi Jain, Hisham Hamed, Kate Haire, Mieke Van Hemelrijck

**Affiliations:** 1 Faculty of Life Sciences and Medicine, Translational Oncology & Urology Research (TOUR), King’s College London, London WC2R 2LS, UK; beth.russell@kcl.ac.uk (B.R.); charlotte.moss@kcl.ac.uk (C.M.); mieke.vanhemelrijck@kcl.ac.uk (M.V.H.); 2 Division of Otolaryngology and Head and Neck Surgery, European Institute of Oncology IRCCS, 20122 Milan, Italy; marta.tagliabue@ieo.it (M.T.); Mohssen.Ansarin@ieo.it (M.A.); 3 Department of Biomedical Sciences, University of Sassari, 07100 Sassari, Italy; 4 South East London Cancer Alliance, London SE1 9RT, UK; harvey.dickinson@gstt.nhs.uk (H.D.); graham.roberts@gstt.nhs.uk (G.R.); sophie.irwin@gstt.nhs.uk (S.I.); Andrew.Davies@gstt.nhs.uk (A.D.); Kate.Haire@gstt.nhs.uk (K.H.); 5 Department of Nephrology and Transplantation, Guy’s & St Thomas’ NHS Foundation Trust, London SE1 9RT, UK; Jonathon.Olsburgh@gstt.nhs.uk; 6 Department of Colorectal Surgery, Guy’s and St Thomas’ NHS Foundation Trust, London SE1 9RT, UK; Francesca.Cocco@gstt.nhs.uk (I.M.F.C.); Alexis.Schizas@gstt.nhs.uk (A.S.); 7 Department of Medical Oncology, Guy’s and St Thomas’ NHS Foundation Trust, London SE1 9RT, UK; Sarah.Mccrindle@gstt.nhs.uk (S.M.); Saoirse.Dolly@gstt.nhs.uk (S.D.); 8 Department of Gynaecological Oncology, Guy’s and St Thomas’ NHS Foundation Trust, London SE1 9RT, UK; Rahul.Nath@gstt.nhs.uk; 9 Department of Otorhinolaryngology Head and Neck Surgery, Guy’s and St Thomas’ NHS Foundation Trust, London SE1 9RT, UK; Aina.Brunet@gstt.nhs.uk (A.B.); Ricard.Simo@gstt.nhs.uk (R.S.); Chrysostomos.Tornari@gstt.nhs.uk (C.T.); 10 Department of Liver Studies, King’s College Hospital, London SE5 9RS, UK; Parthi.Srinivasan@nhs.net (P.S.); Andreas.Prachalias@nhs.net (A.P.); 11 Department of Plastic Surgery, Guy’s and St Thomas’ NHS Foundation Trust, London SE1 9RT, UK; Jenny.Geh@gstt.nhs.uk; 12 Department of Thoracic Surgery, Guy’s and St Thomas’ NHS Foundation Trust, London SE1 9RT, UK; Stephanie.Fraser@gstt.nhs.uk (S.F.); Tom.Routledge@gstt.nhs.uk (T.R.); Rujun.Ma@gstt.nhs.uk (R.M.); 13 Department of Urology, Guy’s and St Thomas’ NHS Foundation Trust, London SE1 9RT, UK; Ella.Doerge@gstt.nhs.uk (E.D.); Ben.Challacombe@gstt.nhs.uk (B.C.); Raj.Nair@gstt.nhs.uk (R.N.); Marios.Hadjipavlou@gstt.nhs.uk (M.H.); 14 Department of Colorectal Surgery, King’s College Hospital, London SE5 9RS, UK; Rosaria.Scarpinata@nhs.net; 15 Department of Colorectal Surgery, Lewisham and Greenwich NHS Trust, London SE13 6LH, UK; Paolo.Sorelli@nhs.net; 16 Division of Urology, European Institute of Oncology IRCCS, 20122 Milan, Italy; FrancescoAlessandro.Mistretta@ieo.it (F.A.M.); Gennaro.Musi@ieo.it (G.M.); Ottavio.deCobelli@ieo.it (O.d.C.); 17 Department of Oncology and Hemato-Oncology, University of Milan, 20122 Milan, Italy; Lorenzo.Spaggiari@ieo.it; 18 Division of Thoracic Surgery, European Institute of Oncology IRCCS, 20122 Milan, Italy; Monica.Casiraghi@ieo.it; 19 Division of Gynaecological Surgery, European Institute of Oncology IRCCS, 20122 Milan, Italy; Alessia.aloisi@ieo.it (A.A.); Andrea.DellAcqua@ieo.it (A.D.); Angelo.Maggioni@ieo.it (A.M.); 20 Division of Data Management, European Institute of Oncology IRCCS, 20122 Milan, Italy; Donatella.Scaglione@ieo.it (D.S.); Stefania.Zanoni@ieo.it (S.Z.); daniele.rampaziodasilva@ieo.it (D.R.D.S.); Daniela.Brambilla@ieo.it (D.B.); Raffaella.Bertolotti@ieo.it (R.B.); Giulia.Peruzzotti@ieo.it (G.P.); 21 Medical Administration, European Institute of Oncology, IRCCS, 20122 Milan, Italy; Fabrizio.Mastrilli@ieo.it; 22 Department of Experimental Oncology, European Institute of Oncology IRCCS, 20122 Milan, Italy; Sara.Gandini@ieo.it; 23 Department of Breast Surgery, Guy’s and St Thomas’ NHS Foundation Trust, London SE1 9RT, UK; Jain.urvashi@gstt.nhs.uk (U.J.); Hisham.hamed@gstt.nhs.uk (H.H.)

**Keywords:** COVID-19, cancer surgery, postoperative outcomes

## Abstract

**Simple Summary:**

A better understanding of the reality for cancer patients during the SARS-CoV-2 (COVID-19) pandemic will help us readapt current prediction models. There is a need to dive into rich data sources from apex cancer centres. The aim of our retrospective study was to report on the outcomes of cancer patients receiving radical surgery with curative intent during the first wave of the COVID-19 pandemic. Data from two cancer centres that were at the epicentre of the outbreak from March to September 2020 (as well as a comparator group in 2019) were utilised for this study. We observed that while there was a decline in number of surgeries performed, the implemented COVID-19 minimal pathways were safe for cancer patients requiring surgical treatment.

**Abstract:**

The SARS-CoV-2 (COVID-19) pandemic is having a large effect on the management of cancer patients. This study reports on the approach and outcomes of cancer patients receiving radical surgery with curative intent between March and September 2020 (in comparison to 2019) in the European Institute of Oncology, IRCCS (IEO) in Milan and the South East London Cancer Alliance (SELCA). Both institutions implemented a COVID-19 minimal pathway where patients were required to self-isolate prior to admission and were swabbed for COVID-19 within 72 h of surgery. Positive patients had surgery deferred until a negative swab. At IEO, radical surgeries declined by 6% as compared to the same period in 2019 (*n* = 1477 vs. 1560, respectively). Readmissions were required for 3% (*n* = 41), and <1% (*n* = 9) developed COVID-19, of which only one had severe disease and died. At SELCA, radical surgeries declined by 34% (*n* = 1553 vs. 2336). Readmissions were required for 11% (*n* = 36), <1% (*n* = 7) developed COVID-19, and none died from it. Whilst a decline in number of surgeries was observed in both centres, the implemented COVID-19 minimal pathways have shown to be safe for cancer patients requiring radical treatment, with limited complications and almost no COVID-19 infections.

## 1. Introduction

The new severe acute respiratory syndrome SARS-CoV-2 (COVID-19) was declared a pandemic on 11 March 2020 by the World Health Organisation [1]. This is why healthcare organisations in many countries across the globe have made many significant changes to their healthcare services aiming to face the challenges of the COVID-19 pandemic, leading to a significant disruption in the provision of cancer treatments [2]. The common aim has been to equip institutions to manage the surge of critical patients, and also to minimise face to face contact where possible to reduce further transmission of the virus. Surgical activity in particular has faced many challenges. In the UK and Ireland on 17 March, the National Health Service (NHS) advised to cancel all non-urgent elective surgeries [2]. About two weeks before, in accordance with the anticipated peak in southern Europe, these restrictive measures were also taken in Italy [3,4]. The Italian National Health System (INHS) interrupted all non-urgent surgeries, outpatient consultations, and rehabilitation services [5]. This was initially intended to free up the necessary resources and enable surgical staff to treat COVID-19 patients. Various studies have reported an important decrease in cancer referrals, creating a concern that many cancer patients had a delay in their diagnosis and treatment, resulting in further advancement of their disease [6,7]. Moreover, a recent meta-analysis found that a delay in surgical cancer treatment of four weeks is associated with a 6–8% increase in the risk of death [8]. Another recent cohort study also reported that substantial increases in the number of avoidable cancer deaths in England are to be expected as a result of diagnostic delays due to the COVID-19 pandemic in the UK [9].

The European Institute of Oncology, IRCCS (IEO) in Milan is one of the largest cancer hospitals in Italy. The South East London Cancer Alliance (SELCA) includes three major hospital trusts: Guy’s and St. Thomas’, Lewisham and Greenwich Trust, and Kings College Hospital. Milan and London were both at the epicentre of the first COVID-19 wave, and their surgical staff were forced to implement strict COVID-19 pathways to continue providing cancer care. However, only few studies have analysed the safety of these pathways.

In the event of another wave of COVID-19, it is important to evaluate the safety of the COVID-19 pathway that was followed for cancer patients requiring radical surgery [10]. Here, we analysed rich data sources from apex Cancer Centres to describe the COVID-19 safety approach and report on the demographic characteristics and surgical outcomes of those cancer patients undergoing radical treatment. This will further inform future clinical guidelines and help readapt current prediction models.

## 2. Materials and Methods

### 2.1. Study Population

At the IEO, the study population consisted of all patients undergoing scheduled radical surgery with curative intent for gynaecological, head and neck (H&N), thoracic and urological cancers between 1 March and 30 September 2020, as well as the comparable period of 1st March to 30th September 2019. Only patients with complete data were included in the study. In southeast London, all patients undergoing scheduled radical surgery for breast, colorectal, liver, plastic/skin, and upper gastrointestinal cancer, in addition to gynaecological, H&N, thoracic and urological cancers were included from 23 March to 8 September 2020, and the comparable period in 2019. The periods observed varied due to the earlier start of the COVID-19 pandemic in Italy.

Data collected from all participants included gender, age, socioeconomic status, ethnicity, comorbidities (hypertension, diabetes mellitus (DM), lung conditions, renal impairment, liver conditions, cardiovascular disease (CVD)), performance status (according to the World Health Organisation (WHO)) [11], body mass index (BMI), tumour site, American Society of Anaesthesiologists (ASA) classification [12], surgery time, theatre time, >24 h of intensive care unit (ICU) stay, length of stay (LOS), readmissions, complications according to the Clavien Dindo Classification [13], post-operative COVID-19 status, and death by any cause. Data on COVID-19 severity (mild/moderate and severe) and COVID-19-related deaths were only available for the IEO study population. The weekly number of COVID-19 cases in London was extracted from the Public Health England Coronavirus dashboard [14]. The weekly number of COVID-19 cases in Milan was extracted from the Italian Ministry of Health portal [15,16].

### 2.2. Patient Pathway—IEO

Since the initial phases of the pandemic, dedicated personnel were in charge of ascertaining the absence of signs/symptoms of COVID-19 and establishing the urgency/priority of outpatient visits. Hospital entry was only allowed for patients. The use of surgical masks was compulsory, and body temperature was measured by infrared thermometers, where only patients with a body temperature under 37.5 degrees Celsius were allowed to enter. Before admission, all patients underwent a telephone triage to assess their current health status, lack of COVID-19 symptoms (fever, cough, flu-like symptoms, anosmia), and possible contact with COVID-19 positive people or those with symptoms indicative of COVID-19. From April 1st 2020, a nasopharyngeal swab for COVID-19 was collected for all head and neck (HNC) and Thoracic patients at pre-surgical assessment; all the other surgical specialties started to perform nasopharyngeal swab pre-surgical assessment from 1 September 2020. With COVID negative patients, surgery was scheduled within 3 to 5 days from the swab. In COVID positive patients, two consecutive negative swabs and 14 days of self-isolation was required to perform surgery.

The anaesthetic protocol was devised to minimise aerosol generation and potential exposure to undetected COVID-19 infection in patients with false negative swab tests. All the involved staff were required to wear full personal protection equipment (PPE) and only the anaesthetist and nurse had access to the operating theatre during the patient’s anaesthetic procedure. During the surgery, all staff involved had to remain wearing full PPE throughout the surgical procedure. In the post-operative period, patients were in single rooms with surgical masks and all visiting healthcare professionals were required to use full PPE when entering the room. Updates on the state and outcome of the patients to family members was given via telephone. All patients with COVID-19 suspected symptoms from all specialties were subjected to chest X-ray and/or computed tomography (CT) and nasopharyngeal swab.

### 2.3. Patient Pathway—SELCA

A multidisciplinary team assessed patients’ risk profiles according to new government guidance in relation to their co-morbidities and the potential negative effects of COVID-19. If the health risks were deemed too high, patient care was directed to an alternative non-surgical pathway. The need for a post-operative critical care unit (CCU) bed was evaluated, and if deemed too high and prolonged, alternative treatments were considered. An enhanced consenting process was utilised, which included agreed levels of care in the postoperative period with some patients electing not to have CCU care if their condition deteriorated after surgery. Similar to the IEO pathway, all patients were instructed to self-isolate for 14-days to minimise the risk of acquiring COVID-19 infection in the pre-operative period. Moreover, two negative swabs were required in surgical pre-assessment in order to proceed to surgery. If the staging computed tomography (CT) scan of the chest identified incidental COVID-19 disease, then surgery would be delayed for 14 days, even if the patients had had two negative swabs.

Prior to the surgery, all patients were intubated in the operating theatre with the anaesthetic team wearing full PPE. Once the endotracheal tube was placed, the surgical team waited 20 min before entering the theatre, this was to allow for adequate air exchanges to occur and minimise the exposure to aerosol. Throughout the surgery, the team was made up of only consultant surgeons, as junior doctors were deployed to other COVID-19 related duties for the first 2 months of the pandemic. Full PPE was adopted by all theatre staff. Another 20 min were taken after the patient was extubated upon completion of the surgical procedure prior to transfer to the recovery room. Additionally, there was a mandatory simulation training programme for all theatre staff, which included putting on and removing PPE techniques, intubation techniques and failed intubation drills.

Full PPE used by all physicians in both Institutes were filtering face piece 3 (FFP3) mask, in addition to a surgical mask, water-repellent disposable gown or apron, double gloves, and protective goggles or visor. In the surgical theatres, the protocol was to have an area of gowning and de-gowning [17,18]. At IEO, also, the health personnel were swabbed every 15 days to detect asymptomatic vectors.

### 2.4. Statistical Analyses

Descriptive statistics were performed to describe baseline socio-demographic and clinical characteristics, surgical, and COVID-19 outcomes. Absolute and relative frequencies for categorical variables, median values, and interquartile (IQR) ranges for continuous variables are reported. Differences in patient characteristics between March to September 2020 and the comparable period in 2019 were evaluated with the Z-score test for two population proportions.

## 3. Results

### 3.1. IEO

At IEO, there were 1477 radical surgeries with curative intent performed from March to September 2020 (270 for gynaecological, 339 for H&N, 377 for thoracic, and 491 for urological cancers), compared to 1560 surgeries in the same period in 2019 (274 for gynaecological, 350 for H&N, 460 for thoracic, and 476 urological cancers). There was a decline of 6% in 2020 compared to 2019. The main decline was seen for thoracic surgery where 18% less surgeries were performed in 2020 compared to 2019 (*p* = 0.01). On the other hand, there was a 3% (*n* = 490 vs. 476) increase in urological cancer surgeries in 2020 compared to 2019 (*p* = 0.10).

Clinical and demographic characteristics were all comparable between both periods for all cancer types (Table A1 and Table 1). Surgical outcomes of IEO patients between 1 March and 30 September 2020 are summarised in Table 2. When looking at ASA grade, 207 (14%) patients had a grade of III or higher (24 (9%) of gynaecological, 60 (18%) of H&N, 81 (21%) of thoracic, and 42 (9%) of urological cancers). The median surgery time was 155 min for all cancers, while the median theatre time was 226 min. Major complications (Clavien Dindo Classification III or higher) were recorded for 3% (*n* = 49). Readmissions were required for 3% (23 (9%) for gynaecological, 14 (4%) for H&N, 2(<1%) for thoracic, and 2 (<1%) for urological cancers). ICU stay of more than 24 h was required for 5% (*n* = 78) of all cancer patients (8 (3%) of gynaecological, 1 (<1%) for H&N, 68 (18%) of thoracic, and 1 (<1%) for urological cancers.

Nine (1%) patients developed COVID-19 post-operatively (1 (<1%) of gynaecological, 7 (2%) of H&N, and 1(<1%) of urological cancer); of these, only the gynaecological patient went on to develop severe disease and died from COVID-19. Death from other causes was seen in 11 (1%) of cancer patients (2(<1%) of gynaecological, 3 (1%) of H&N, and 6 (2%) of thoracic cancers). Table 3 summarises COVID-19 outcomes in IEO patients.

**Figure 1:**
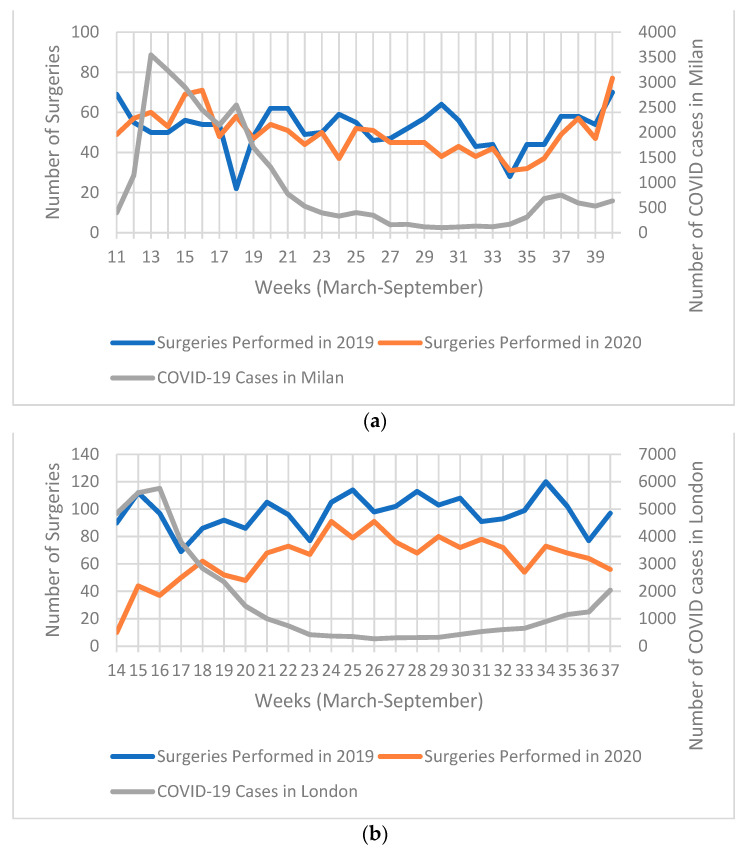
Chart illustrating: (**a**) weekly COVID-19 cases in Milan (and metropolitan area), and number of surgeries 1 March 2020–30 September 2020; (**b**) weekly SARS-CoV-2 (COVID-19) cases in London (and metropolitan area), and number of surgeries performed in South East London Cancer Alliance (SELCA) between 23 March 2019–8 September 2019 and 23 March 2020–8 September 2020.

Figure 1a illustrates the number of weekly COVID-19 cases in Milan and surgeries per week for 1 March to 30 September 2020, as well as the comparable period in 2019. There was no constant marked decline in the number of surgeries performed in 2020 compared to the same period in 2019, except for weeks 27 to 32 where less surgeries were performed in 2020. The number of COVID-19 cases had a steep rise during the first four weeks observed, reaching 3545 cases in week 13 (15–21 March). Subsequently, the number of weekly COVID-19 cases began to gradually decrease with the exception of a second smaller peak in week 18 (2546 cases from 20th to 26th March). However, from week 35 onwards, cases began to moderately rise again.

### 3.2. SELCA

At SELCA centres in London, there was a decline of 34% radical surgeries performed from 23 March to 8 September 2020, compared to the same period in 2019. There were 1553 radical surgeries in the observed period (321 of breast, 129 of colorectal, 114 of gynaecological, 152 of H&N, 92 of liver, 56 of plastics, 305 thoracic, 72 of upper gastrointestinal (GI), and 312 of urological cancers), compared to 2336 in 2019. The most notable declines were seen for plastic/skin surgeries, with a decline of 80% (*n* = 56 vs. 278, *p* = 0.00); followed by colorectal with 59% (*n* = 129 vs. 310, *p* = 0.00), and breast with 38% (*n* = 321 vs. 519, *p* = 0.24). However, there was an increase in the number of H&N (9%, *n* = 152 vs. 139, *p* = 0.00) and upper GI (18%, *n* = 72 vs. 61, *p* = 0.00) cancer surgeries in 2020 compared to 2019.

Clinical and demographic characteristics are shown in Table A1, Table 4 and Table 5. Most of these characteristics were comparable between both periods, except for performance status, where more patients with lower performance status were operated on in 2020, compared to 2019.

Surgical outcomes for SELCA patients 23 between March and 8 September 2020 are summarised in Table 2. A total of 240 patients (22%) had an ASA grade of III or higher (12 (4%) of breast, 18 (14%) of colorectal, 19 (17%) of gynaecological, 20 (13%) of H&N, 12 (13%) of liver, 11 (20%) of plastic, 91 (30%) of thoracic, 9 (13%) of upper GI, and 48 (15%) of urological cancers. Median surgery and theatre times were 120 and 195 min, respectively for all cancers. A total of 36 (11%) of cancer patients required readmission (2 (1%) of breast, 5 (4%) of colorectal, 8 (5%) of H&N, 6 (7%) of liver, 1 (2%) of plastic, 6 (2%) of thoracic, and 2 (1%) of urological cancers). As for time in the ICU, 155 (11%) of cancer patients required stays of more than 24 h (19 (6%) of breast, 50 (39%) of colorectal, 3 (18%) of gynaecological, 47 (31%) of H&N, 68 (74%) of liver, 1 (2%) of plastic, 152 (50%) of thoracic, 44 (61%) of upper GI, and 42 (13%) of urological cancers). A total of 55 (6%) of patients developed pneumonia, and 52 (17%) were from thoracic surgery.

Of the total of patient undergoing radical surgery, 7 (<1%) developed COVID-19 (1 (<1%) of breast, 1 (<1%) of gynaecological, 4 (3%) of H&N, and 1(<1%) of urological cancers. No patients died from COVID-19 complications. A total of 27 (2%) of patients died of other causes (2 (1%) of H&N, 2 (2%) of liver, 1 (2%) of plastic, 15 (5%) of thoracic, and 7 (3%) of urological cancers), where only 6 (<1%) died within 30 days. COVID-19 outcomes for cancer patients undergoing radical surgery are summarised in Table 3.

Figure 1b illustrates the number of weekly COVID-19 cases in London and surgeries per week for 23rd March to 8th September 2020, as well as the comparable period in 2019. There was a significant decrease in the number of surgeries throughout the observed period. The biggest difference was seen in the first 3 weeks analysed (23 March to 12 April). During these first three weeks observed, the number of COVID-19 cases were at their highest, reaching 5760 cases in week 16 (6–12 March). The number of surgeries in 2020 began to rise from week 17 (13 April); however, they never reached the same number as 2019. On the other hand, COVID-19 cases began to decline from week 17 onwards and maintained a plateau until cases began to rise again starting week 30 (13 July 2020).

## 4. Discussion

The IEO in Milan and SELCA hospitals in London were both at the epicentre of the first COVID-19 wave. While a decline of 6% and 34% in number of surgeries was observed in Milan and London, respectively, the current study has shown that the implemented COVID-19 minimal pathways are safe for cancer patients requiring radical treatment. Even with different geographical setting, patient characteristics were comparable between both cancer hubs except for performance status, where in the SELCA population, more surgeries were performed in patients with a lower performance status (0 and 1). This may have been to avoid patients with higher risk of developing severe COVID-19 disease and complications from getting infected. Regarding COVID-19 and surgical outcomes, readmissions in IEO were required for 3% (*n* = 41) and 3% (*n* = 59) had major complications. Less than 1% (*n* = 9) developed COVID-19, of which 80% (*n* = 7) were H&N cancers and had a mild/moderate disease. Only one gynaecological cancer patient had severe disease and died. In SELCA, readmissions were required for 11% (*n* = 36) and <1% (*n* = 7) developed COVID-19 in the post-operative period. Details on the severity and/or death from COVID-19 were not reported. Only 2% (*n* = 27) died from any cause.

Regardless of the high increase in the number of COVID-19 cases, the IEO saw a small decline in oncological surgery overall. Similar results were reported by Maspero et al. where cancer surgery was prioritised and remained stable, while overall surgical activity saw up to an 84% reduction in volume, both with minimal mortality due to COVID-19 [19]. In the IEO population, the biggest decline in number of surgeries was seen for thoracic cancers (18%, *n* = 377 vs. 460), this may be due to more restrictions applied in the selection of patients with underlying lung conditions, as these patients have been reported to have an increased risk of developing severe COVID-19 disease [20]. Moreover, a decline in the number of surgeries may reflect a gap in the early diagnosis of cancer due to COVID-19. On the other hand, the number of urological surgeries increased by 3% (*n* = 491 vs. 476) in 2020 compared to 2019; this was not in line with previous studies, where urological surgeries overall (including oncological surgeries) had a decline in volume of surgeries during the COVID-19 pandemic [21,22]. The IEO was chosen by the region as a reference centre for other non-operative hospitals for pandemic urgency. Many urological cancer patients were sent from these centres to the IEO, and this may explain the increase in this number for this division. In addition, the pathway undertaken by the IEO in Milan is similar to other pathways described in other Italian centres, which have had similar favourable results [23].

In the UK, cancer services and especially aggressive tumour types were given high priority to continue their activity (i.e., thoracic, H&N, and upper GI) [24]. In our SELCA population, overall, oncological surgery had a decline of 34% (*n* = 1553 vs. 2336) in 2020 compared with 2019. The largest declines were seen for plastic/skin (80%, *n*= 56 vs. 278), colorectal (59%, *n* = 129 vs. 310), and breast (38%, *n* = 321 vs. 519) cancer, while H&N and upper GI had an increase of 9% and 18%, respectively (*n* = 152 vs. 139, *n* = 72 vs. 61). The increase in upper GI surgeries may be explained by an increase in minimally invasive surgeries for cancer patients and by a higher number of emergency procedures (bleeding/perforation) performed in the upper GI tract [25,26]. The large decrease in number of breast surgeries is likely due to delayed breast cancer diagnoses and a lower number of breast screening tests performed during this period. Similar results were seen in another observational study [27]. Moreover, the large decrease in number of surgeries for “non-urgent” cancer may have been due to the outbalance of high risk of new contagions compared with the risk of cancer progression in many cases. Similar results were reported by various cancer centres where surgeries were cancelled or postponed for months [24,28]. However, after the first wave of the pandemic, the number of cancer surgeries began to rise gradually [29]. Additionally, several studies have now reported on safe pathways to perform “non-urgent” cancer surgeries with minimal complications due to COVID-19 [30,31,32,33].

Overall, in both cancer hubs, the decision-making for the surgical prioritisation was individually reviewed by a panel of clinicians and dedicated virtual tumour board, and treatment plans were personalised taking into account patient comorbidities, performance status, tumour characteristics, availability of other oncological treatments, etc. The risk and benefits of each surgical procedure should be thoroughly weighed against the potential spread of COVID-19 disease [34]. Thus, an individualised approach is imperative in the treatment of cancers during the COVID-19 pandemic [18]. Moreover, the cancellation and postponement of elective and “non-urgent” cancer surgeries has created a backlog of patients who were planned to undergo radical treatment; this has great implications for both patients and healthcare providers [23]. It is critical for more cancer centres and specialties to begin to implement similar pathways in their institutes to reintroduce elective cancer surgery to prevent unnecessary delays in patient care.

Alarming results were reported by a large international survey that highlighted insufficient pre-operative screening of COVID-19 in the current surgical practice [28,35,36]. In addition, this survey found several discrepancies on the various pathways implemented across cancer centres. Thus, it is important to further inform future clinical guidelines to install a universal safe pathway to treat cancer patients. Further epidemiological studies are needed comparing data for more cancer sites, as well as cancer types and types of surgeries performed to have a more detailed look into the safest pathways for cancer patients. In addition, it is important for future studies to describe criteria used for choosing patients who are apt to undergo oncological surgery. Lastly, studies on the effects of surgical cancellation, on both patients and healthcare staff, are much needed to analyse the true effect the COVID-19 pandemic had on cancer care.

To our knowledge, the current study is among the first large cohorts comparing safe pathways for oncological surgery implemented in various hospitals in Milan and south-east London. Both of these cities were among the hardest-hit cities in Europe, and thus, they were among the first centres to implement COVID-19 minimal pathways. One of the major limitations of our study was the lack of data on COVID-19 severity status for SELCA patients.

## 5. Conclusions

Our findings suggest that although cancer patients have been previously identified as high risk from COVID-19, the implemented COVID-19 minimal pathways described here are safe for patients who require radical treatment. The implementation of hospital prevention plans designed to avoid the entry of COVID-19 positive patients and healthcare professionals are essential to safeguard delicate oncologic patients already hospitalised, the health personnel, and to be able to continue the life-saving activity of cancer centres. It is critical for all elective cancer surgeries to go back to their normal levels of functioning to avoid future complications due to delays in oncological care.

## Figures and Tables

**Table 1:** European Institute of Oncology, IRCCS (IEO) patient characteristics of cancer patients receiving radical surgery between 1 March 2019–30 September 2019 and 1 March 2020–30 September 2020, divided by cancer site.

	Head and Neck			Gynaecological			Urological			Thoracic		
	2019 (*n* = 350, %)	2020 (*n* = 339, %)	*p*-Value	2019 (*n* = 274, %)	2020 (*n* = 270, %)	*p*-Value	2019 (*n* = 476%)	2020 (*n* = 491, %)	*p*-Value	2019 (*n* = 460, %)	2020 (*n* = 377, %)	*p*-Value
Difference (%)	−4%			−1%			+3%			−18%		
Sex												
Male	210 (60)	215 (63)	0.35	0	0		431 (91)	445 (91)	0.96	214 (47)	192 (51)	0.43
Female	140 (40)	124 (37)	0.35	274 (100)	270 (100)		45 (9)	46 (9)	0.96	246 (53)	185 (49)	0.43
Age												
<50	76 (22)	74 (22)	0.97	62 (23)	51 (19)	0.28	24 (5)	24 (5)	0.91	53 (12)	45 (12)	0.000
50–59	66 (19)	57 (17)	0.48	72 (26)	69 (26)	0.84	114 (24)	122 (25)	0.74	88 (19)	77 (20)	0.03
60–69	92 (26)	86 (25)	0.78	79 (29)	75 (28)	0.78	201 (42)	207 (42)	0.98	154 (33)	102 (28)	0.62
70–79	90 (26)	91 (27)	0.73	51 (19)	61 (22)	0.25	128 (27)	129 (26)	0.82	137 (29)	121 (32)	0.16
≥80	26 (7)	31 (9)	0.41	10 (3)	14 (5)	0.38	9 (2)	9 (2)	0.94	28 (7)	32 (8)	0.44
Mean (SD ^1^)	61 (14.5)	61 (15.1)	0.84	59 (12.8)	60.5 (13.1)	0.80	63.6 (8.8)	64 (8.6)	0.88	64 (12)	64 (13.1)	0.41
Socio-economic Status												
Low	101 (29)	120 (35)	0.06	45 (16)	34 (13)	0.20	36 (8)	25 (5)	0.11	45 (10)	33 (9)	0.05
Medium	97 (28)	141 (42)	0.000	70 (26)	56 (21)	0.18	113 (24)	96 (20)	0.11	110 (24)	81 (21)	0.23
High	138 (39)	60 (18)	0.000	148 (54)	146 (54)	0.98	273 (57)	297 (60)	0.32	273 (59)	201 53)	0.56
Missing	14 (4)	18 (5)	0.41	11 (4)	34 (13)	0.000	54 (11)	73 (15)	0.10	32 (7)	62 (16)	0.81
Ethnicity												
White British	0	0	0	0	1 (<1)	0.31	0	0		0	0	
White Other	346 (99)	335 (99)	0.96	268 (99)	263 (99)	0.75	474	490 (99)	0.54	458 (99)	374 (99)	0.12
Black Caribbean	0	1 (<1)	0.31	0	0		0	0		0	0	
Black African	0	0	0	0	0		1 (<1)	0	0.31	0	0	
Black Other	1 (<1)	1 (<1)	0.98	0	0		1 (<1)	1 (<1)	0.98	0	0	
Asian	1 (<1)	2 (<1)	0.54	4 (<1)	3 (<1)	0.71	0	0		2 (<1)	1 (<1)	0.01
Mixed	0	0		0	0		0	0		0	2 (<1)	0.48
Other	2 (<1)	0	0.15	2 (<1)	3 (<1)	0.64	0	0		0	0	
Unknown	0	0		0	0		0	0		0	0	
Co-morbidities												
Hypertension	141 (40)	113 (33)	0.05	74 (27)	76 (28)	0.76	184	180 (37)	0.52	167 (36)	130 (34)	0.06
Diabetes Mellitus	27 (8)	35 (10)	0.23	27 (10)	8 (<1)	0.000	39 (39)	36 (7)	0.61	45 (10)	15 (4)	0.76
Lung Conditions	26 (7)	24 (7)	0.85	11 (4)	6 (<1)	0.22	21 (5)	18 (4)	0.55	34 (8)	43 (11)	0.61
Renal Impairment	8 (2)	5 (1)	0.43	5 (2)	1 (<1)	0.10	7 (1)	11 (2)	0.37	14 (3)	2 (<1)	0.72
Liver Conditions	11 (3)	6 (2)	0.24	6 (2)	3 (<1)	0.32	11 (2)	13 (3)	0.73	8 (2)	2 (<1)	0.55
CVD ^2^	57 (16)	63 (19)	0.42	30 (11)	24 (9)	0.42	82 (17)	74 (15)	0.36	95 (21)	62 (16)	0.52
Performance status												
0	182 (52)	212 (63)	0.004	239 (87)	261 (97)	0.000	NA	NA		NA	NA	
1	162 (46)	120 (35)	0.003	28 (10)	8 (3)	0.000	NA	NA		NA	NA	
2	5 (<1)	7 (2)	0.52	6 (2)	0	0.01	NA	NA		NA	NA	
3	1 (<1)	0	0.31	1 (<1)	1 (<1)	0.99	NA	NA		NA	NA	
4	0	0		0	0		NA	NA		NA	NA	

^1^ Standard deviation, ^2^ Cardiovascular disease.

**Table 2:** Surgical outcomes of IEO and SELCA patients receiving radical treatment from between 1 March 2020–30 September 2020 and 23 March 2020–8 September 2020.

	Breast (*n*,%)	Colorectal (*n*,%)	Gynaecological (*n*,%)	Head and Neck (*n*,%)	Liver (*n*,%)	Plastics (*n*,%)	Thoracic (*n*,%)	Upper Gastrointestinal (*n*,%)	Urology (*n*,%)	Total (*n*,%)
IEO										
Surgeries			*n* = 270	*n* = 339			*n* = 377		*n* = 491	*n* = 1477
ASA ^1^ grade III/IV/V			24 (9)	60 (18)			81 (21)		42 (9)	207 (14)
Surgery time—mins (Median, IQR ^2^)			191 (28–658)	112 (8–777)			101 (54–161)		214 (70–525)	155 (23–687)
Theatre time—mins (Median, IQR)			274 (59–733)	163 (23–949)			178 (117–242)		288 (121–639)	226 (50–787)
ICU ^3^ stay >24 h			8 (3)	1 (<1)			68 (18)		1 (<1)	78 (5)
Pneumonia			6 (2)	4 (1)			0		2 (<1)	12 (1)
LOS ^4^—days			4	4			5		3	4
Re-admissions			23 (9)	14 (4)			2 (<1)		2 (<1)	41 (3)
Complications			58 (21)	64 (19)			57 (15)		43 (9)	222 (15)
I			18 (7)	39 (12)			23 (6)		12 (2)	92 (6)
II			20 (7)	8 (2)			25 (7)		28 (6)	81 (6)
IIIA			5 (2)	1 (<1)			6 (2)		1 (<1)	13 (<1)
IIIB			8 (3)	14 (4)			0		2 (<1)	24 (2)
IVA			5 (2)	1 (<1)			1 (<1)		0	7 (<1)
IVB			0	1 (<1)			1 (<1)		0	2 (<1)
V			2 (1)	0			1 (<1)		0	3 (<1)
SELCA										
Surgeries	*n* = 321	*n* = 129	*n* = 114	*n* = 152	*n* = 92	*n* = 56	*n* = 305	*n* = 72	*n* = 312	*n* = 1553
ASA grade III/IV/V	12 (4)	18 (14)	19 (17)	20 (13)	12 (13)	11 (20)	91 (30)	9 (13)	48 (15)	240 (22)
Surgery time—mins (Median, IQR)	79 (55–106)	143 (90–218)	149 (100–192)	126 (74–330)	217 (159–320)	50 (39–111)	118 (85–148)	187 (116–292)	145 (55–190)	120 (73–183)
Theatre time—mins (Median, IQR)	140 (115–173)	237 (175–332)	232 (184–290)	194 (141–445)	309 (255–413)	112 (69–175)	195 (161–225)	290 (208–411)	201 (113–254)	195 (138–263)
ICU stay >24 h	19 (6)	50 (39)	3 (18)	47 (31)	68 (74)	1 (2)	152 (50)	44 (61)	42 (13)	155 (11)
Pneumonia	0	0	0	2 (1)	0	0	52 (17)	0	0	55 (6)
LOS—days	1	6	1	3	7	0	6	8	2	4
Re-admissions	2 (1)	5 (4)	9	8 (5)	6 (7)	1 (2)	6 (2)	0	2 (1)	36 (11)

^1^ American Society of Anesthesiologists, ^2^ Interquartile range, ^3^ Intensive care unit, ^4^ Length of stay.

**Table 3:** COVID-19 outcomes of IEO and SELCA patients receiving radical treatment from between 1 March 2020–30 September 2020 and 23 March 2020–8 September 2020.

	Breast *n*, %	Colorectal *n*, %	Gynaecological *n*, %	Head and Neck *n*, %	Liver *n*, %	Plastics *n*, %	Thoracic *n*, %	Upper Gastrointestinal *n*, %	Urology *n*,%	Total *n*, %
IEO										
Surgeries			*n* = 270	*n* = 339			*n* = 377		*n* = 491	*n* = 1477
COVID status										
Negative			176 (65)	332 (98)			288 (76)		490 (99)	1286 (87)
Positive			1 (<1)	7 (2)			0		1 (<1)	9 (1)
Unknown			93 (34)	0			89 (24)		0	182 (12)
COVID severity										
Mild and moderate			0	7 (2)			0		1 (<1)	8 (<1)
Severe			1 (<1)	0			0		0	1 (<1)
Death										
All-cause (30 days)			2 (<1)	3 (1)			6 (2)		0	11 (1)
All-cause (90 days)			1 (<1)	0			0		0	1 (<1)
SELCA										
Surgeries	*n* = 321	*n* = 129	*n* = 114	*n* = 152	*n* = 92	*n* = 56	*n* = 305	*n* = 72	*n* = 312	*n* = 1553
COVID status										
Negative										
Positive	1 (<1)	0	1 (<1)	4	0	0	0	0	1 (<1)	7 (<1)
Unknown										
Death										
All-cause (30 days)	0	0	0	0	0	0	4 (1)	0	2 (1)	6 (<1)
All-cause (90 days)	0	0	0	2 (1)	2 (2)	1 (2)	11 (4)	0	5 (2)	21 (1)

**Table 4:** SELCA patient characteristics of cancer patients receiving radical surgery between 23 March 2019–8 September 2019 and 23 March 2020–8 September 2020, divided by cancer site.

	Breast			Colorectal			Gynaecological			Head and Neck		
	2019 (*n* = 519, %)	2020 (*n* = 321, %)	*p*-Value	2019 (*n* = 310, %)	2020 (*n* = 129, %)	*p*-Value	2019 (*n* = 171, %)	2020 (*n* = 114, %)	*p*-Value	2019 (*n* = 139, %)	2020 (*n* = 152, %)	*p*-Value
Difference (%)	−38%			−59%			−33%			+9%		
Sex												
Male	6 (1)	6 (2)	0.42	174 (56)	71 (55)	0.83	0	0		71 (51)	75 (49)	0.38
Female	513 (99)	315 (98)	0.42	136 (44)	58 (45)	0.83	171 (100)	114 (100)		68 (49)	77 (51)	0.38
Age												
<50	163 (31)	116 (36)	0.16	34 (11)	16 (12)	0.67	41 (24)	35 (31)	0.21	44 (32)	49 (32)	0.45
50–59	33 (6)	97 (30)	0.00	49 (16)	33 (26)	0.02	13 (8)	31 (27)	0.00	9 (8)	37 (24)	0.00
60–69	145 (25)	58 (18)	0.00	49 (16)	38 (29)	0.00	47 (27)	23 (20)	0.14	29 (21)	38 (25)	0.20
70–79	110 (21)	38 (12)	0.00	88 (28)	28 (22)	0.13	30 (18)	19 (17)	0.84	31 (22)	24 (16)	0.07
≥80	68 (13)	12 (4)	0.00	90 (29)	14 (11)	0.00	40 (23)	6 (5)	0.00	26 (19)	4 (3)	0.00
Mean (SD ^1^)	56 (13.3)	54 (12.9)		68 (13.7)	64 (13.1)		59 (14.8)	57 (14.4)		59 (14.7)	57 (16.2)	
Socioeconomic Status												
Low	101 (19)	59 (18)	0.69	44 (14)	20 (16)	0.72	32 (19)	15 (13)	0.20	22 (16)	27 (18)	0.32
Medium	289 (56)	171 (54)	0.49	118 (39)	73 (57)	0.00	93 (54)	66 (58)	0.55	76 (55)	77 (51)	0.24
High	108 (21)	91 (28)	0.01	88 (28)	36 (27)	0.91	46 (27)	33 (29)	0.70	39 (28)	47 (31)	0.29
Missing	21 (4)	0	0.00	60 (19)	0	0.00	0	0		2 (1)	1 (<1)	0.25
Ethnicity												
White British	165 (32)	91 (28)	0.28	86 (28)	34 (26)	0.76	49 (29)	34 (30)	0.83	72 (53)	58 (38)	0.009
White Other	52 (10)	42 (13)	0.18	25 (8)	10 (8)	0.91	22 (13)	17 (15)	0.62	15 (11)	14 (9)	0.32
Black Caribbean	28 (5)	18 (6)	0.89	4 (1)	1 (<1)	0.60	2 (1)	2 (2)	0.69	5 (4)	2 (1)	0.10
Black African	16 (3)	12 (4)	0.61	8 (3)	0	0.00	5 (3)	6 (5)	0.34	3 (2)	3 (2)	0.45
Black Other	28 (5)	17 (5)	0.95	2 (1)	4 (3)	0.12	5 (3)	6 (5)	0.34	1 (<1)	5 (3)	0.05
Asian	23 (4)	11 (3)	0.45	2 (1)	4 (3)	0.12	5 (3)	2 (2)	0.51	9 (6)	7 (5)	0.24
Mixed	15 (3)	11 (3)	0.66	0	2 (2)	0.15	0	0		2 (1)	0	0.07
Other	8 (2)	8 (2)	0.35	2 (1)	0	0.15	4 (2)	3 (2)	0.87	1 (<1)	2 (1)	0.30
Unknown	184 (35)	111 (35)	0.79	181 (57)	74 (57)	0.84	79 (46)	44 (39)	0.20	31 (22)	61 (41)	0.00
Comorbidities												
Hypertension	57 (11)	20 (6)	0.01	29 (9)	2 (2)	0.00	53 (31)	10 (9)	0.00	46 (33)	3 (2)	0.00
Diabetes Mellitus	25 (5)	11 (3)	0.31	17 (5)	8 (6)	0.77	27 (16)	6 (5)	0.00	22 (56)	1 (<1)	0.00
Lung Conditions	4 (1)	6 (2)	0.19	24 (8)	6 (5)	0.19	3 (2)	3 (3)	0.62	7 (5)	4 (3)	0.14
Renal Impairment	8 (2)	0	0.00	17 (5)	2 (2)	0.01	14 (8)	1 (<1)	0.00	5 (4)	0	0.01
Liver Conditions	1 (<1)	0	0.31	9 (3)	2 (2)	0.34	3 (2)	0	0.08	1 (<1)	1 (<1)	0.47
CVD ^2^	4 (1)	6 (2)	0.19	8 (3)	11 (9)	0.02	9 (5)	1 (<1)	0.02	8 (6)	2 (1)	0.02
Performance status												
0	134 (26)	185 (58)	0.00	21 (7)	41 (32)	0.00	16 (9)	42 (37)	0.00	6 (4)	32 (21)	0.00
1	134 (26)	81 (25)	0.84	48 (15)	40 (31)	0.00	85 (50)	52 (46)	0.49	41 (34)	49 (32)	0.30
2	47 (9)	0	0.00	15 (5)	9 (7)	0.40	45 (26)	6 (5)	0.00	27 (19)	9 (6)	0.00
3	2 (<1)	1 (<1)	0.85	2 (1)	1 (<1)	0.88	8 (5)	3 (3)	0.35	1 (<1)	0	0.15
4	14 (3)	1 (<1)	0.00	0	0		1 (<1)	4 (4)	0.10	0	0	
Unknown	188 (36)	19 (6)	0.00	224 (72)	38 (29)	0.00	16 (9)	7 (6)	0.30	64 (27)	62 (41)	0.18

^1^ Standard deviation, ^2^ Cardiovascular disease.

**Table 5:** FSELCA patient characteristics of cancer patients receiving radical surgery between 23 March 2019–8 September 2019 and 23 March 2020–8 September 2020, divided by cancer site.

	Liver			Plastics			Thoracic			Upper Gastrointestinal			Urology		
	2019 (*n* = 116, %)	2020 (*n* = 92, %)	*p*-Value	2019 (*n* = 278, %)	2020 (*n* = 56, %)	*p*-Value	2019 (*n* = 342, %)	2020 (*n* = 305, %)	*p*-Value	2019 (*n* = 61, %)	2020 (*n* = 72, %)	*p*-Value	2019 (*n* = 400, %)	2020 (*n* = 312, %)	*p*-Value
Difference (%)	−20%			−80%			−10%			+18%			−22%		
Sex															
Male	76 (66)	44 (48)	0.00	143 (51)	40 (71)	0.00	160 (47)	128 (42)	0.11	42 (69)	53 (74)	0.54	330 (83)	242 (78)	0.00
Female	40 (34)	48 (52)	0.00	135 (49)	16 (29)	0.00	182 (53)	177 (58)	0.06	19 (31)	19 (26)	0.54	70 (17)	70 (22)	0.00
Age															
<50	14 (12)	12 (13)	0.41	70 (25)	8 (14)	0.04	15 (4)	22 (7)	0.00	4 (7)	5 (7)	0.92	56 (14)	49 (16)	0.00
50–59	11 (9)	16 (17)	0.04	60 (22)	13 (23)	0.79	38 (11)	40 (13)	0.21	3 (5)	23 (32)	0.00	48 (12)	83 (27)	0.00
60–69	24 (21)	31 (34)	0.01	52 (19)	4 (7)	0.00	50 (15)	100 (33)	0.23	8 (13)	22 (31)	0.01	100 (25)	88 (28)	0.00
70–79	31 (27)	27 (29)	0.33	51 (18)	15 (27)	0.18	88 (26)	117 (38)	0.00	19 (31)	18 (25)	0.43	110 (28)	63 (20)	0.00
≥80	36 (31)	6 (7)	0.00	45 (16)	16 (29)	0.05	151 (44)	26 (9)	0.00	27 (44)	4 (5)	0.00	86 (22)	29 (9)	0.00
Mean (SD ^1^)	67 (12.7)	65 (11.5)		64 (17.9)	70 (15.3)		71 (10.9)	69 (10.5)		67 (10.6)	62 (10.2)		63 (14.7)	63 (14.3)	
Socioeconomic Status															
Low	8 (7)	4 (4)	0.21	14 (5)	10 (18)	0.01	51 (15)	40 (13)	0.40	11 (18)	10 (14)	0.51	48 (12)	59 (19)	0.00
Medium	34 (29)	48 (52)	0.00	115 (41)	20 (36)	0.42	162 (47)	133 (44)	0.21	21 (34)	35 (49)	0.09	178 (45)	148 (47)	0.00
High	32 (28)	40 (43)	0.00	107 (38)	26 (46)	0.27	127 (37)	132 (43)	0.01	27 (44)	27 (38)	0.42	151 (38)	101 (32)	0.00
Missing	42 (36)	0	0.00	42 (15)	0	0.00	2 (1)	0	0.82	2 (3)	0	0.15	23 (6)	4 (1)	0.00
Ethnicity															
White British	23 (20)	24 (26)	0.14	90 (32)	23 (41)	0.22	96 (28)	49 (16)	0.00	29 (48)	25 (35)	0.13	79 (20)	75 (2)	0.13
White Other	5 (4)	1 (1)	0.06	17 (6)	3 (5)	0.82	17 (5)	16 (5)	0.82	5 (8)	4 (6)	0.55	24 (6)	28 (9)	0.14
Black Caribbean	2 (2)	3 (3)	0.24	1 (<1)	0	0.31	1 (<1)	3 (1)	0.76	2 (3)	0	0.15	9 (2)	6 (2)	0.13
Black African	1 (1)	1 (1)	0.43	0	0		2 (<1)	2 (1)	0.86	0	3 (4)	0.07	10 (3)	8 (3)	0.02
Black Other	0	0		1 (<1)	0	0.31	3 (<1)	2 (1)	0.70	1 (2)	1 (1)	0.90	17 (4)	9 (3)	0.00
Asian	0	0		0	9	0.00	0	2 (1)		2 (3)	0	0.15	5 (1)	0	0.04
Mixed	3 (3)	0	0.03	0	1 (2)	0.31	2 (<1)	0	0.33	0	1 (1)	0.31	1 (<1)	2 (<1)	0.00
Other	6 (5)	1 (1)	0.03	0	0		2 (<1)	1 (<1)	0.61	0	2 (3)	0.15	4 (1)	1 (<1)	0.00
Unknown	76 (65)	62 (67)	0.38	169 (61)	29 (52)	0.21	219 (64)	230 (75)	0.00	22 (36)	36 (50)	0.10	251 (63)	183 (59)	0.00
Comorbidities															
Hypertension	2 (2)	14 (15)	0.00	22 (8)	10 (18)	0.06	156 (46)	23 (8)	0.00	32 (52)	0	0.00	115 (29)	8 (3)	0.00
Diabetes Mellitus	2 (2)	9 (10)	0.00	9 (3)	3 (5)	0.50	51 (15)	22 (7)	0.00	7 (11)	0	0.00	46 (12)	21 (7)	0.00
Lung Conditions	1 (1)	18 (20)	0.00	4 (1)	3 (5)	0.20	0	32 (10)		7 (11)	2 (3)	0.05	9 (2)	14 (4)	0.00
Renal Impairment	0	0		3 (1)	0	0.08	25 (7)	1 (<1)	0.00	3 (5)	0	0.07	67 (17)	3 (1)	0.00
Liver Conditions	0	5 (5)	0.01	0	0		5 (1)	0	0.12	4 (7)	0	0.03	6 (2)	0	0.16
CVD ^2^	1 (1)	22 (24)	0.00	5 (2)	10 (18)	0.00	33 (10)	37 (12)	0.08	4 (7)	0	0.03	13 (3)	21 (7)	0.00
Performance status															
0	1 (1)	47 (51)	0.00	2 (1)	19 (34)	0.00	13 (4)	62 (20)	0.21	1 (2)	8 (11)	0.01	85 (21)	146 (47)	0.00
1	8 (7)	18 (20)	0.00	5 (2)	4 (7)	0.13	47 (14)	75 (25)	0.00	15 (25)	26 (36)	0.14	69 (17)	46 (15)	0.00
2	2 (2)	14 (15)	0.00	6 (2)	4 (7)	0.16	10 (3)	11 (4)	0.42	5 (8)	3 (4)	0.34	21 (5)	13 (4)	0.00
3	0	0		0	1 (2)	0.31	2 (<1)	0	0.33	0	0		1 (<1)	2 (<1)	0.15
4	0	0		0	1 (2)	0.31	1 (<1)	0	0.49	0	0		0	0	0.5
Unknown	105 (91)	13 (14)		265 (95)	27 (48)	0.00	269 (79)	157 (51)	0.00	40 (65)	35 (49)	0.04	224 (56)	105 (34)	0.00

^1^ Standard deviation, ^2^ Cardiovascular disease.

## Data Availability

The data presented in this study are available on request from the corresponding author. The data are not publicly available due to ethical reasons.

## Appendix A

**Table A1:** Contains the patient characteristics of all cancer patients receiving radical surgery between 1 March 2019–30 September 2019 and 1 March 2020–30 September 2020 at the IEO, and 23 March 2019–8 September 2019 and 23 March 2020–8 September 2020 at SELCA.

	IEO			SEL Cancer Alliance		
	2019 (*n* = 1560, %)	2020 (*n* = 1477, %)	*p*-Value	2019 (*n*= 2336, %)	2020 (*n* = 1553, %)	*p*-Value
Difference (%)	−6%			−34%		
Sex						
Male	855 (55)	852 (58)	0.11	1003 (43)	659 (42)	0.75
Female	705 (45)	625 (42)	0.11	1333 (57)	894 (58)	0.75
Age						
<50	215 (14)	194 (13)	0.60	441 (19)	312 (20)	0.35
50–59	340 (22)	325 (22)	0.88	264 (11)	373 (24)	0.000
60–69	526 (34)	470 (32)	0.26	504 (22)	402 (26)	0.002
70–79	406 (26)	402 (32)	0.45	558 (24)	349 (23)	0.30
≥80	73 (5)	86 (6)	0.15	569 (24)	117 (7)	0.000
Socioeconomic Status						
Low	227 (15)	212 (14)	0.87	331 (14)	244 (16)	0.18
Medium	390 (25)	374 (25)	0.83	1086 (46)	771 (49)	0.05
High	832 (53)	704 (48)	0.001	725 (31)	533 (34)	0.03
Missing	111 (7)	187 (13)	0.000	194 (8)	5 (1)	0.00
Ethnicity						
White British	0	1	0.31	689 (29)	413 (27)	0.04
White Other	1546 (99)	1462 (99)	0.73	182 (8)	135 (9)	0.31
Black Caribbean	0	1 (<1)	0.31	54 (2)	35 (2)	0.90
Black African	1 (<1)	0	0.31	45 (2)	35 (2)	0.48
Black Other	2 (<1)	2 (<1)	0.95	58 (2)	44 (3)	0.50
Asian	7 (<1)	6 (<1)	0.85	46 (2)	26 (2)	0.49
Mixed	0	2 (<1)	0.15	23 (1)	17 (1)	0.74
Other	4 (<1)	3 (<1)	0.75	27 (1)	28 (2)	0.10
Unknown	0	0		1212 (52)	830 (52)	0.33
Co-morbidities						
Hypertension	566 (36)	499 (34)	0.14	512 (22)	90 (13)	0.000
Diabetes Mellitus	138 (9)	94 (6)	0.009	206 (9)	81 (12)	0.000
Lung Conditions	92 (6)	91 (6)	0.76	59 (3)	88 (13)	0.000
Renal Impairment	34 (2)	19 (1)	0.05	142 (6)	7 (1)	0.000
Liver Conditions	36 (2)	24 (2)	0.17	29 (1)	8 (1)	0.01
CVD ^1^	177 (11)	223 (15)	0.002	85 (4)	110 (16)	0.000
Performance status						
0	1357 (87)	1341 (91)	0.000	279 (30)	582 (53)	0.000
1	190 (12)	128 (9)	0.001	452 (48)	391 (36)	0.000
2	11 (<1)	7 (<1)	0.40	178 (19)	103 (9)	0.23
3	2(<1)	1(<1)	0.59	16 (2)	8 (<1)	0.49
4	0	0		16 (2)	6 (<1)	0.19

^1^ Cardiovascular disease.
